# K^+^-Dependent Photocycle and Photocurrent Reveal the Uptake of K^+^ in Light-Driven Sodium Pump

**DOI:** 10.3390/ijms241914414

**Published:** 2023-09-22

**Authors:** Jikang Xu, Qifan Yang, Baofu Ma, Longjie Li, Fei Kong, Lan Xiao, Deliang Chen

**Affiliations:** 1 College of Life Sciences, University of Chinese Academy of Sciences, Beijing 100049, Chinamabaofu@dicp.ac.cn (B.M.);; 2 National Laboratory of Biomacromolecules, Institute of Biophysics, Chinese Academy of Sciences, Beijing 100101, China

**Keywords:** microbial rhodopsins, light-driven Na^+^ pumps, ion transport

## Abstract

Engineering light-controlled K^+^ pumps from Na^+^-pumping rhodopsins (NaR) greatly expands the scope of optogenetic applications. However, the limited knowledge regarding the kinetic and selective mechanism of K^+^ uptake has significantly impeded the modification and design of light-controlled K^+^ pumps, as well as their practical applications in various fields, including neuroscience. In this study, we presented K^+^-dependent photocycle kinetics and photocurrent of a light-driven Na^+^ pump called *Nonlabens dokdonensis* rhodopsin 2 (NdR2). As the concentration of K^+^ increased, we observed the accelerated decay of M intermediate in the wild type (WT) through flash photolysis. In 100 mM KCl, the lifetime of the M decay was approximately 1.0 s, which shortened to around 0.6 s in 1 M KCl. Additionally, the K^+^-dependent M decay kinetics were also observed in the G263W/N61P mutant, which transports K^+^. In 100 mM KCl, the lifetime of the M decay was approximately 2.5 s, which shortened to around 0.2 s in 1 M KCl. According to the competitive model, in high KCl, K^+^ may be taken up from the cytoplasmic surface, competing with Na^+^ or H^+^ during M decay. This was further confirmed by the K^+^-dependent photocurrent of WT liposome. As the concentration of K^+^ increased to 500 mM, the amplitude of peak current significantly dropped to approximately ~60%. Titration experiments revealed that the ratio of the rate constant of H^+^ uptake (k_H_) to that of K^+^ uptake (k_K_) is >10^8^. Compared to the WT, the G263W/N61P mutant exhibited a decrease of approximately 40-fold in k_H_/k_K_. Previous studies focused on transforming NaR into K^+^ pumps have primarily targeted the intracellular ion uptake region of *Krokinobacter eikastus* rhodopsin 2 (KR2) to enhance K^+^ uptake. However, our results demonstrate that the naturally occurring WT NdR2 is capable of intracellular K^+^ uptake without requiring structural modifications on the intracellular region. This discovery provides diverse options for future K^+^ pump designs. Furthermore, we propose a novel photocurrent-based approach to evaluate K^+^ uptake, which can serve as a reference for similar studies on other ion pumps. In conclusion, our research not only provides new insights into the mechanism of K^+^ uptake but also offers a valuable point of reference for the development of optogenetic tools and other applications in this field.

## 1. Introduction

Microbial rhodopsins are hepta-helical transmembrane photoreceptive proteins with diverse functions, such as ion pumps, ion channels, sensors, and enzymes [1,2,3]. The first microbial rhodopsin discovered was a light-driven H^+^ pump bacteriorhodopsin (BR) from *Halobacterium salinarum* [4]. In the over half-century since then, numerous microbial rhodopsins have been identified in all three domains of life, most of which are light-driven H^+^ pumps or Cl^−^ pumps [5,6].

In 2013, *Krokinobacter eikastus* rhodopsin 2 (KR2), found in marine bacteria, was reported [7]. It belongs to a novel family of Na^+^-pumping microbial rhodopsin (NaR) [8,9,10,11,12,13]. KR2 binds an all-*trans* retinal via a Schiff base linked to a lysine in the seventh helix [14,15] and exhibits a unique NDQ (Asn112-Asp116-Gln123) motif in the third helix, homologous to the DTD (Asp85-Thr89-Asp96) motif in BR and the TSA (Thr126-Ser130-Ala137) motif in halorhodopsin (HR) [16,17]. Upon photoexcitation, all-*trans* retinal isomerizes to the 13-*cis* form, leading to a K intermediate [18,19,20,21,22], followed by the equilibrium of L and M intermediates [7,20,23,24]. The Schiff base proton is transferred to Asp116 upon the formation of M intermediate [7,14,25,26,27], which reduces the energy barrier to Na^+^ transport [28]. Upon the formation of an O intermediate, Na^+^ is recruited into the cytoplasmic ion-selectivity cavity consisting of Gly263 and Asn61 [14,15] and is then transported to its binding site in the vicinity of the Schiff base counterion region [11,29]. Finally, Na^+^ is released to the extracellular surface during the decay of the O intermediate to the ground state [23,30,31,32]. KR2 pumps Na^+^ outward under physiological conditions but works as a H^+^ pump in the absence of Na^+^ or at low pH [7]. To elucidate the selective ion transport, the competitive uptake of Na^+^ and H^+^ has been proposed [33].

Many ion-pumping microbial rhodopsins, including archaerhodopsin and HR, have been used as optogenetic tools to silence neuronal activity [34]. When excited, however, they may cause unphysiological intracellular pH changes or Cl^−^ accumulation. In contrast, the outward transport of Na^+^ or K^+^ evokes noninvasive hyperpolarization of membrane potential. Thus, NaR and K^+^-pumping rhodopsins appear to be the next-generation optogenetic tool for neural silencing [14,35,36,37,38]. In particular, a K^+^ pump would be highly desirable, as K^+^ is the main ion used for neuronal polarization. Furthermore, they actively manipulate intracellular K^+^ concentration, unlike passive K^+^ channels that rely on electrochemical potential, which may expand the scope of optogenetic applications. Thus far, natural microbial rhodopsins that actively pump K^+^ have not been discovered. This obstacle significantly hinders the widespread application of light-controlled K^+^ in fields such as neuroscience. Therefore, it becomes particularly important to design K^+^-pumping rhodopsins based on NaR. 

Natural NaR pumps Na^+^ or smaller monovalent cations, like Li^+^, but appears to exclude K^+^ and other ions larger than Na^+^ [7,39]. When the cytoplasmic ion uptake cavity was modified, the transport activity of K^+^ or even Cs^+^ was reported in KR2 [14,39]. However, the crystal structure of mutant G263F, which can pump K^+^, has demonstrated a dramatically reduced ion uptake cavity rather than an enlarged one as would be expected to enable K^+^ permeability [40]. Nevertheless, to engineer K^+^-pumping rhodopsin, mutations in the intracellular ion-uptake cavity, like G263W, G263F, and N61Y, all showed K^+^-pumping activity, which was further increased in G263W/N61P [14]. S254A mutated near the Schiff base region also exhibited K^+^ pump activity [40]. In addition, the transport of K^+^ was reported in N-terminal modified KR2 (eKR2) [37]. However, few kinetic studies on the transport of K^+^ have been reported, and the mechanism of K^+^ selectivity in NaR remains unclear. The limited knowledge regarding the kinetic and selective mechanism of K^+^ uptake has significantly impeded the design of light-driven K^+^ pumps, as well as their practical applications.

In this study, we focused on the photocycle kinetics of *Nonlabens dokdonensis* rhodopsin 2 (NdR2, also known as DDR2 or NQ rhodopsin) in KCl [8,41,42]. To understand the selective uptake of K^+^ by NdR2, we monitored the M intermediate kinetics at various concentrations of K^+^ or Na^+^ using flash photolysis. As a NaR, NdR2 shares about 77% sequence identity with KR2. The characteristic NDQ motif and many other residues involved in Na^+^ pumping are well conserved. Photolysis and FTIR studies have displayed similar spectral features between KR2 and NdR2 [41,43]. Previous studies have primarily modified the intracellular ion uptake region of KR2 to enhance K^+^ uptake. However, our novel results of the K^+^-dependent photocycle and photocurrent in the wild type (WT) NdR2, demonstrate that the naturally occurring NdR2 is capable of intracellular K^+^ uptake without requiring structural modifications. This discovery indicates that NdR2 serves as a promising candidate upon which the future design of the K^+^ pump is based. Furthermore, distinct from the conventional activity assay of ion transport, we introduced the kinetic analysis of competitive ion uptake and the analysis of photocurrent into the study of K^+^ uptake. Our results together with the innovative approach not only contribute to a better understanding of the mechanisms underlying K^+^ uptake and selectivity but also provide methodological assistance and inspiration for applications such as screening efficient K^+^ pumps and the engineering of microbial rhodopsins. 

## 2. Results

### 2.1. K^+^-Dependent Photocycle Kinetics

**Figure 1:**
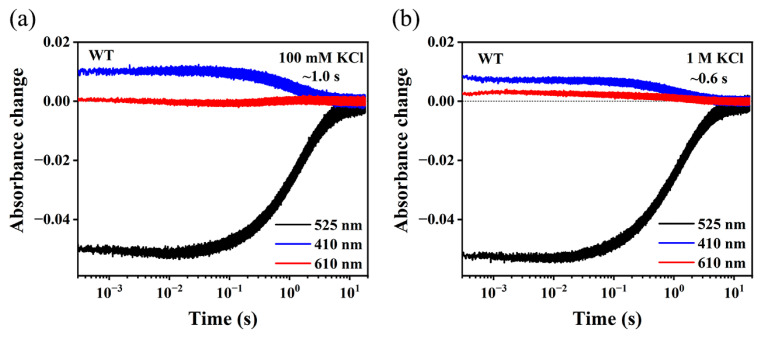
Photocycle kinetics of WT NdR2 at pH 8.0. Absorbance changes of the WT in 0.05% DDM Scheme. (**a**) 100 mM KCl and (**b**) 1 M KCl are shown at characteristic wavelengths of 525 nm, 410 nm, and 610 nm. The apparent lifetime of M intermediate is indicated.

In the H^+^-pumping photocycle of both lipid-reconstituted and detergent-solubilized NaR, a dominant accumulation of M intermediate was present. When Na^+^ was transported, however, M decay was accelerated by approximately three orders of magnitude [7,41,44]. As a kinetic consequence, the accumulation of M intermediate was suppressed, while a significant O intermediate was present. In this study, the kinetics of the M and O intermediates were investigated by monitoring the absorbance changes detected at 410 nm and 610 nm, respectively. The apparent lifetimes of the M state were determined by fitting the kinetics trace at 410 nm using exponential fitting. Consistent with previously published results, the DDM-solubilized NdR2 in 100 mM NaCl (Figure S1a) shows an M intermediate decay with a lifetime of sub-milliseconds and a significant accumulation of O intermediate. In contrast to its Na^+^-pumping photocycle, NdR2 (Figure 1a) shows a dominant M intermediate with a much slower lifetime (~1.0 s) detected at 410 nm in 100 mM KCl, a putative H^+^-pumping condition [7,44]. The intermediate of O was hardly detected at 610 nm. In 1 M KCl (Figure 1b), however, we detected an accelerated decay of M intermediate with an apparent lifetime of 0.6 s compared with the decay of M in 100 mM KCl. Correspondingly, the amount of accumulated M intermediate was reduced. An increase in the absorbance change at 610 nm was detected over the time range from a few sub-milliseconds to several seconds, which could reflect the kinetics of both K and O intermediates. We also observed an acceleration of M decay kinetics in 1 M KBr (Figure S1b), consistent with the trend observed in KCl (Figure 1b). In 1 M NMG^+^ (Figure S1c), an organic cation that is not transported by NaR, however, we did not observe the characteristic accelerated M decay as that in 1 M KCl. Instead, Figure S1c shows a typical H^+^-pumping photocycle with a slower M decay. This excludes possible artifacts due to the high ion strength of 1 M K^+^ solutions. Additionally, the characteristic absorption spectrum of NdR2 in 1 M KCl is identical to that in 100 mM KCl (Figure S2a), showing the absorption maximum at 525 nm. This eliminates the possibility that the photocycle changes in 1 M KCl may be caused by the shift of the absorption peak. Therefore, the results above suggest that the characteristic photocycle kinetics in highly concentrated KCl were related to K^+^.

**Figure 2:**
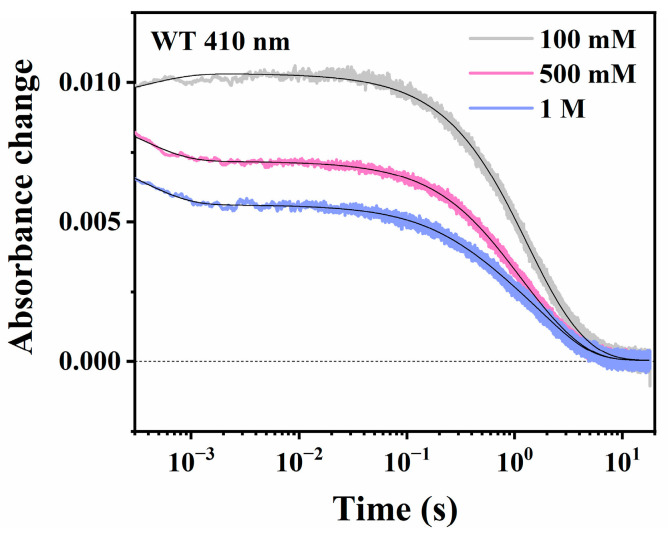
K+-dependent M kinetics of WT NdR2 at pH 8.0. Absorbance changes of the WT in 100 mM, 500 mM, and 1 M KCl, containing 0.05% DDM, were measured at 410 nm. Exponential fitting lines are shown in black.

During the decay of the M intermediate, Na^+^ is taken up from the cytoplasmic surface to the Schiff base cavity [7]. The increase in the concentration of substrate Na^+^, therefore, results in the acceleration of M decay [9,33]. In WT NdR2, we also observed the [K^+^] dependence of M intermediate decay. Figure 2 shows M decay kinetics in 100 mM, 500 mM, and 1 M KCl, respectively, demonstrating an apparent acceleration of M decay. Kinetic analysis (Table 1) reveals two major decay components using exponential fitting, including a K^+^-dependent M decay component (τ_1_) and a K^+^-independent M decay component (τ_2_). It appears that the lifetime of the faster component decreases as [K^+^] increases, while that of the slower component hardly changes. NdR2 only pumps H^+^ and presents one slower M decay (~2.1 s) in NMG^+^ solution; we, therefore, attribute this slower component to H^+^ transport. Since the H^+^-pumping activity of WT NdR2 dominates in 100 mM or even higher concentrations of KCl (Section 2.2), this slower component is always present (Table 1). On the other hand, the faster decay component responds to the change in [K^+^]. In line with the Na^+^ dependence of M decay in the Na^+^-pumping condition, the K^+^ dependence of this faster decay component indicates that the characteristic photocycle in solutions with high K^+^ concentration might be associated with K^+^ uptake. As shown in Figure S3, this faster component of M decay is further confirmed by its corresponding rise of O intermediate in 500 mM KCl. It has a rise lifetime of ~0.7 s, which matches the lifetime of the corresponding K^+^-dependent M decay component (~0.6 s) (Table 1). A small fraction of delayed K intermediate before the rise of O intermediate was also detected at 610 nm. In 1 M KCl, due to the increase in this delayed K intermediate, the rise of the O intermediate could not be ambiguously resolved (Figure 1b). Nevertheless, the K^+^-dependent M decay component (τ_1_) and K^+^-independent M decay component (τ_2_) revealed by exponential fitting indicate that K^+^ uptake may occur in WT NdR2, in addition to H^+^ uptake.

**Figure 3:**
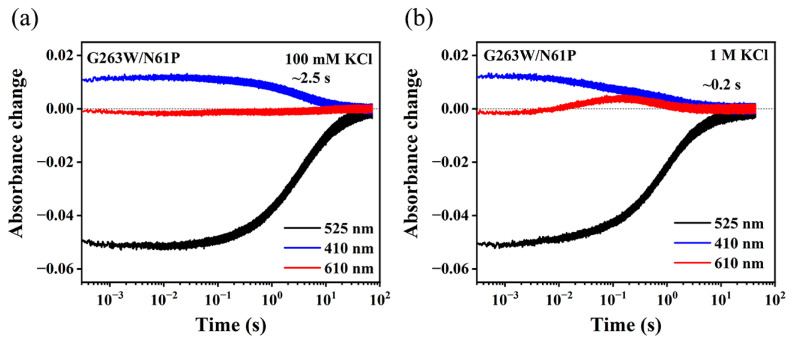
Photocycle kinetics of G263W/N61P at pH 8.0. Absorbance changes of G263W/N61P in 0.05% DDM solution, containing (**a**) 100 mM KCl and (**b**) 1 M KCl, are shown at characteristic wavelengths of 525 nm, 410 nm, and 610 nm. The apparent lifetime of M intermediate in KCl is indicated.

Additionally, the kinetics of the G263W/N61P mutant, which transports K^+^ in KR2 [14], also shows the accelerated M decay in 1M KCl, relative to that in 100 mM KCl (Figure 3). As a kinetic consequence, the accelerated transition from M to O intermediate leads to an increase in the amplitude of O intermediate. Relative to the WT, this mutant shows a less delayed K intermediate and a more prominent O intermediate, which is likely due to a more pronounced change in M decay. Nevertheless, these differences did not impact the findings that both the WT and the putative K^+^-pumping G263W/N61P exhibited a K^+^-dependent M decay kinetics, which confirmed the correlation between the characteristic photocycle kinetics in the WT and the uptake of K^+^. Namely, WT NdR2 may also take up K^+^ under high concentrations of K^+^. Like WT, the characteristic absorption spectrum of G263W/N61P in 1 M KCl was identical to that in 100 mM KCl (Figure S2b), excluding that the kinetic changes in 1 M KCl may have resulted from the possible shift of absorption peak.

### 2.2. K^+^-Dependent Photocurrent

**Figure 4:**
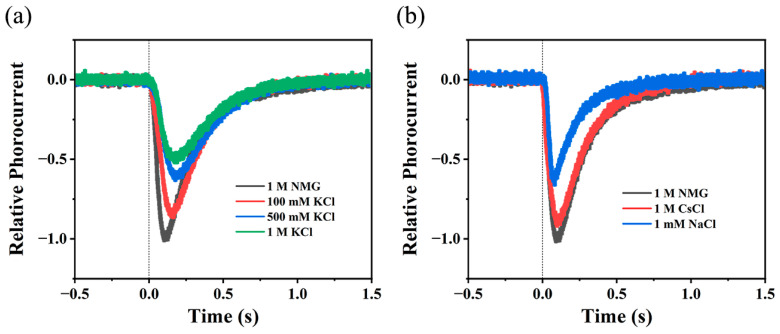
Transient photoelectric response of WT NdR2 using protein-based photocurrent assay at pH 8.0. (**a**) K^+^-dependent photocurrent compared to the signal detected in 1 M NMG-Cl. (**b**) Photocurrent in 1 mM NaCl and 1 M CsCl solution, compared to the signal detected in 1 M NMG-Cl. The continued green light was used to generate the transient photoelectric response. The vertical dotted line indicates the switching on the illumination. All the measurements were performed under identical ionic strength conditions using NMG-Cl as background salt solution if needed.

The photoelectric response has been widely used to investigate the ion transport in microbial rhodopsins. Under continuous illumination, the typical transient photocurrent of the BR-coated ITO electrode reflects the transient uptake/release of H^+^ at the protein surface [45,46,47]. Similarly, we immobilized lipid-reconstituted WT NdR2 on the surface of ITO glass that was reportedly sensitive to the change in [H^+^] [47,48]. As shown in Figure 4a, switching on the green light induced a transient negative photocurrent signal in 1 M NMG^+^ solution, a H^+^-pumping condition. The negative polarity indicates that the uptake of H^+^ precedes the release of H^+^ at the surface of WT NdR2. This is consistent with the sequence of ion uptake/release reported in several NaRs [30,31,44]. As expected, Figure 4b shows that the amplitude of the photocurrent signal significantly decreases in 1 mM NaCl when the transport of Na^+^ and H^+^ coexist. Due to the competitive transport of H^+^ and Na^+^ [33], the uptake of Na^+^ would inhibit the uptake of H^+^, which is then reflected by the decrease in the transient photocurrent. Interestingly, we also observed an evident decrease in the maximal peak current intensity as the concentration of K^+^ increased (Figure 4a). Compared to that in the NMG^+^ solution, the amplitude of peak current in 500 mM KCl significantly drops to ~60%. It appears that K^+^, like Na^+^, may compete with H^+^, which therefore leads to the reduction in H^+^ uptake. On the other hand, we did not observe Cs^+^-dependent photoelectric response. Even in 1 M CsCl (Figure 4b), the photocurrent hardly changes relative to that in 1 M NMG^+^ solution. This is in line with the fact that natural NaR does not transport large cations like Cs^+^ or NMG^+^. In conclusion, photocurrent changes in response to K^+^ support that WT NdR2 uptakes K^+^ in high KCl.

**Figure 5:**
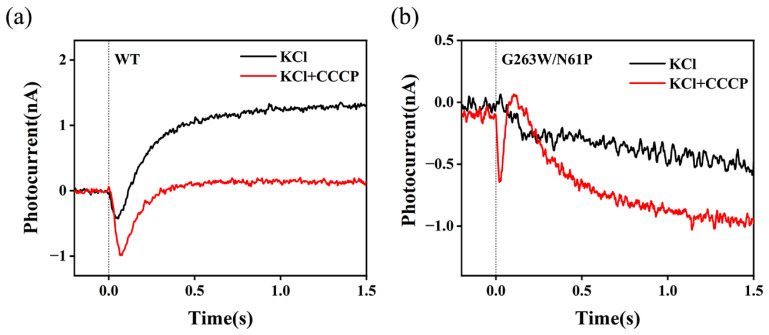
*E. coli* cell-based assay of pump activity. The *E. coli* cells expressing (**a**) WT NdR2 and (**b**) G263W/N61P were equilibrated with 500 mM KCl solution containing 5 mM Tris-HCl at pH 8.0. The experiments were performed in the absence of CCCP and the presence of 50 μM CCCP, respectively. The vertical dotted line indicates turning on the green light.

One may note that the concentration of K^+^ has to reach 500 mM or even higher to achieve an equivalent inhibition produced by 1 mM NaCl. Under low Na^+^ concentrations, such as 1 mM Na^+^ or lower, NdR2 predominantly functions as a proton pump, while the Na^+^ transport activity is too weak to be detected. This suggests that the K^+^ transport activity may also be too subtle to be detected. Indeed, this was confirmed by the *E. coli*-based assay of apparent pump activity. As shown in Figure 5a, we detected a positive stationary current signal in *E. coli* cells expressing WT NdR2 under continuous illumination. In the presence of 50 μM CCCP (CCCP is a mobile proton carrier), this DC signal is significantly suppressed. When H^+^ is the primary ion actively transported outward, the bulk pH decreases. This produces a positive stationary photocurrent, which will be abolished by the addition of CCCP [7,48]. Thus, the WT predominantly pumps H^+^ over K^+^ in 500 mM KCl. In contrast, the mutant G263W/N61P which has significantly improved activity of K^+^ transport in KR2 [14], shows an opposite photocurrent signal. In Figure 5b, we detected a negative stationary photocurrent, which was enhanced by the addition of CCCP. This is because the electrical potential produced by the dominant transport of K^+^ outward drives H^+^ to the surface of the cell membrane, causing an increase in the bulk pH. This is reflected by a negative stationary photocurrent upon illumination. H^+^-uncoupler CCCP facilitates the permeability of H^+^, thereby enhancing the photocurrent.

### 2.3. Kinetic Analysis of K^+^/Na^+^/H^+^ Uptake and Ion Selectivity

**Figure 6:**
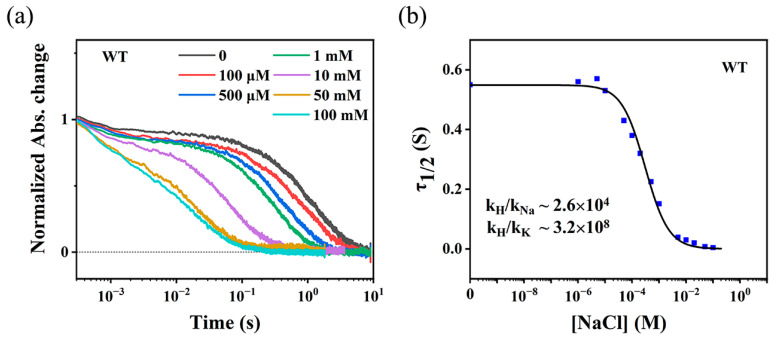
Kinetic analysis of competitive uptake of ions during M decay of WT NdR2 at pH 8.0. The WT in 1 M KCl solution was titrated with 4 M NaCl. (**a**) Normalized absorbance changes at various concentrations of NaCl detected at 410 nm. (**b**) The τ_1/2_ of M intermediate against NaCl concentrations (0–100 mM). The solid curve represents the fitting result corresponding to Equation (2). Calculated values of k_H_/k_Na_ and k_H_/k_K_ are shown accordingly.

According to the kinetics analysis by Kato et al. [33], we monitored the M intermediate kinetics of WT NdR2 and G263W/N61P mutant to evaluate the ability of K^+^ or Na^+^ uptake (Section 4.2). We first titrated the WT sample in 1 M KCl and pH 8.0 solution, with NaCl (Figure 6a), where the uptake of Na^+^ would compete with that of K^+^ and H^+^. As the concentration of Na^+^ increased, the acceleration of M decay was observed accordingly. The τ_1/2_ of M decay plotted against the concentration of NaCl is shown in Figure 6b, which was fitted with Equation (2) (Section 4.2). The resulting curve shows a typical sigmoidal shape of a titration experiment. The ratio of the rate constants k_H_/k_Na_ and k_H_/k_K_, which reflect the relative Na^+^ and K^+^ selectivity during the ion uptake, are about 2.6 × 10^4^ and 3.2 × 10^8^, respectively. This explains why a noticeable acceleration of M decay was not observed at NaCl concentrations below 10 μM (Figure 6b). The reason is that the value of k_Na_[Na^+^], which reflects the apparent reaction rate of Na^+^ uptake is much smaller compared to the apparent rates of H^+^ uptake (k_H_[H^+^]) and K^+^ uptake (k_K_[K^+^]) under conditions of 1 M KCl and pH 8.0. As k_Na_[Na^+^] increases relative to k_H_[H^+^] and k_K_[K^+^] at NaCl concentrations above 10 μM, the competitive uptake of Na^+^ starts to significantly affect the M decay.

To confirm these results, we then titrated the WT sample in 1 M NMG-Cl and pH 8.0 solution with NaCl (Figure S4a,b), where the uptake of Na^+^ would only compete with that of H^+^. The calculated value of k_H_/k_Na_ is about 2.0 × 10^4^, which is in line with the value of ~2.6 × 10^4^ shown in Figure 6b. Given the value of k_H_/k_K_ (~3.2 × 10^8^), the apparent rate of H^+^ uptake (k_H_[H^+^]) should be much larger than that of K^+^ uptake (k_K_[K^+^]), even under high KCl conditions (>100 mM) at neutral pH. This explains why WT NdR2 predominantly transports H^+^ over K^+^ in KCl [41,44], and confirms the results of Figure 4 and Figure 5. Additionally, the confirmed value of k_H_/k_Na_ (~2.6 × 10^4^) explains why NdR2 transports Na^+^ over H^+^ in 100 mM Na^+^ solution at neutral pH, because k_Na_[Na^+^] is approximately 38 times larger than k_H_[H^+^]. 

**Figure 7:**
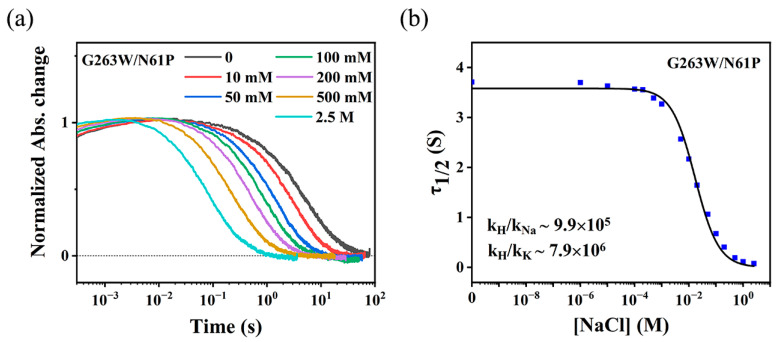
Kinetic analysis of competitive uptake of ions during M decay of G263W/N61P at pH 8.0. The mutant in 50 mM KCl solution was titrated with 4 M NaCl. (**a**) Normalized absorbance changes at various concentrations of NaCl detected at 410 nm. (**b**) The τ_1/2_ of M intermediate against NaCl concentrations (0–2.5 M). The solid curve represents the fitting result corresponding to Equation (2). Calculated values of k_H_/k_Na_ and k_H_/k_K_ are shown accordingly.

According to the analysis of Kato et al. [33], the rate constant of ion uptake may be related to the ion selectivity of NaR. Thus, we propose that a mutant with increased K^+^ transport activity should exhibit a smaller value of k_H_/k_K_ relative to WT NdR2. To verify this, we chose the G263W/N61P mutant, which showed the most efficient transport activity of K^+^ in KR2 studies [14]. This mutant of NdR2 in 50 mM KCl was titrated with NaCl at pH 8.0, where the competitive transport of K^+^/Na^+^/H^+^ could take place. We observed an acceleration of M decay as the concentration of NaCl increased (Figure 7a). The τ_1/2_ of M decay plotted against the concentration of NaCl presents a typical sigmoidal-shaped titration curve (Figure 7b). The value of k_H_/k_Na_ was estimated to be about 9.9 × 10^5^, and the value of k_H_/k_K_ was estimated to be about 8.0 × 10^6^. These values were confirmed by the direct titration experiments with NaCl (k_H_/k_Na_ ~ 7.5 × 10^5^, Figure S5) and with KCl (k_H_/k_K_ ~ 5.5 × 10^6^, Figure S6), respectively. Note that we first titrated G263W/N61P in 1 M KCl using NaCl, similar to the experiments performed on the WT. However, we only obtained a fraction of the sigmoidal-shaped titration dataset with an increase in Na^+^ concentration, this may be attributed to the much smaller k_H_/k_K_ of G263W/N61P (8.0 × 10^6^) compared to that of WT (~3.2 × 10^8^), indicating the mutant has a significantly stronger ability to uptake K^+^ than the WT. As a result, 1 M of KCl for the G263W/N61P sample would be excessively high relative to the WT, leading to significant inhibition of changes in the M decay kinetics. Therefore, we decreased the concentration of KCl to 50 mM, which allowed us to achieve significant changes in M decay and obtain a sigmoidal-shaped curve. Overall, this K^+^-pumping mutant shows an approximately 40-fold decrease in k_H_/k_K_ relative to the WT, which confirms our hypothesis that the increased pump activity of K^+^ may be related to the decrease in k_H_/k_K_. Interestingly, the value of k_H_/k_Na_ in G263W/N61P is approximately 38 times higher than WT, indicating the selectivity for Na^+^ reduces when the ion uptake filter is altered.

## 3. Discussion

### 3.1. The Mechanism of K^+^ Uptake

Previous studies on the pump activity of both WT KR2 and NdR2 in KCl only showed a dominant H^+^-pumping activity by measuring the bulk pH of *E. coli* cells expressing NaR [7,41,44]. When the cytoplasmic ion uptake cavity was modified, the transport activity of K^+^ was reported in KR2 [14,15,39]. Thus far, most assays of the apparent pump activity have suggested that natural NaR may strictly exclude K^+^ and other ions larger than Na^+^ [7,39]. In this study, we are the first to report a K^+^-dependent photocycle and photocurrent of WT NdR2, which have not been previously documented in studies on the NaR family members. Additionally, we propose that K^+^ should be able to be recruited by NdR2 even though the cytoplasmic ion uptake cavity is not modified, indicating that NdR2 may serve as a promising candidate among the NaR family members for the future development of the light-driven K^+^ pump.

In the traditional cell-based assay, the possible flow of K^+^ generated in high KCl might be interfered with by the numerous intrinsic ion channels and pumps embedded in the membrane of *E. coli* cells [12]. To overcome the interference of other proteins, we also tried to reconstitute NdR2 into a liposome and measure its bulk pH. However, the proteoliposomes collapsed and precipitated after being equilibrated in high KCl due to its intrinsic instability under the condition of high ion strength [44]. Finally, we succeeded in immobilizing lipid-reconstituted NdR2 onto the ITO electrode and collected K^+^-dependent transient photocurrent signals. This protein-based photocurrent assay has been widely used to characterize H^+^ uptake or release in many microbial rhodopsin pumps, like BR and HR [45,46,47,48]. The transient current signal triggered by light is very sensitive to the change in H^+^ concentration on the surface of proteins, enabling us to observe the photocurrent changes in response to K^+^ (Figure 4) that have not been reported in the NaR family. Since ITO electrodes can only respond to changes in H^+^ concentration, the observed changes in the apparent photocurrent under NaCl or KCl conditions are not directly generated by variations in Na^+^ or K^+^ concentrations. Instead, these changes are a result of the competitive inhibition of H^+^ uptake by these ions, leading to a reduction in the magnitude of the photocurrent originating from H^+^. 

KR2 takes up H^+^ much more efficiently than Na^+^ under the same concentration of ions. It, however, actually pumps Na^+^ dominantly under physiological conditions because the concentration of Na^+^ is about 10^7^ times higher than that of H^+^ in the ocean [49]. A model of competitive transport of ions has been proposed to explain the ion transport and selectivity in KR2 [9,33]. In high KCl, we also observed the changes in the kinetics of WT NdR2, especially its M decay kinetics which is coupled to the ion uptake. As the concentration of K^+^ increases, the acceleration of M decay (Figure 2 and Table 1) and the inhibition of the H^+^ uptake signal (Figure 4) suggest that K^+^ also may compete with H^+^ during M to O transition. As evidenced by the case of K^+^, our results further support the model of competitive transport.

Although the framework of competitive ion transport has been established, the ion transport mechanisms among the members of the NaR family may vary from one another. *Gillisia limnaea* rhodopsin (GLR) stands out for its ability to pump Na^+^, but not H^+^. Balashov et al. discovered that GLR exhibits an H^+^ release followed by an uptake in NaCl, and a complex H^+^ circulation of release–uptake–release in KCl [9]. They hypothesized that in GLR, the circulating H^+^ at the extracellular side may kinetically compete with Na^+^ uptake at the cytoplasmic side. In contrast, the well-known KR2 can pump both Na^+^ and H^+^ [7]. Kato et al. proposed that the competitive uptake of Na^+^ and H^+^ should occur at the cytoplasmic side [33]. These substrate ions are initially taken up from the cytoplasmic side during the formation of the O intermediate and then released to the extracellular side during the O decay [31]. NdR2, similar to KR2, has been reported in our previous article [44] to pump both Na^+^ and H^+^. The pyranine assay revealed that H^+^ uptake precedes its release, consistent with the photocurrent results obtained in our current study (Section 2.2). We speculate that the functional differences between GLR and NdR2 may stem from variations in the amino acid sequences of the proteins (Figure S7). But, the specific residue responsible for these functional variations remains unclear.

It is well known that the overall apparent photocycle of KR2 in NaCl is composed of the population of H^+^-dependent photocycle and that of Na^+^-dependent photocycle [9,33]. In conditions of low Na^+^ concentration, which favors H^+^-pumping, the overall kinetics of KR2 is predominantly influenced by the feature of H^+^-dependent photocycle. Consequently, the expected acceleration of M decay in response to changes in [Na^+^] does not appear to be evident [33]. According to the competitive ion uptake model, the populations of H^+^-dependent photocycle and K^+^-dependent photocycle should co-exist in KCl. Because the WT NdR2 exhibits a predominant H^+^-pumping activity (Figure 5a) in high KCl, the overall kinetics, including M decay, therefore, are mainly influenced by the feature of H^+^-dependent photocycle (a dominant and slower M decay). This may significantly mask the feature of K^+^-dependent photocycle (a faster M decay in response to [K^+^]). This may explain why in our study the acceleration of M decay did not look so significant when comparing the results obtained with 100 mM and 1 M KCl (Figure 1).

M and O intermediates of KR2 appear sequentially in the Na^+^-pumping photocycle. When the Schiff base is re-protonated upon the M decay, Na^+^ binds to the Schiff base cavity, which generates the red-shifted O intermediate [7]. Because the apparent kinetics of M decay is mainly influenced by the feature of the H^+^-dependent population with a slower M decay of ~2.0 s in high KCl, the feature of K^+^-dependent M decay may visually be overshadowed and lead to the erroneous conclusion that K^+^-dependent M decay is slower than its O-decay. Nevertheless, we resolved a K^+^-dependent M decay component of ~0.6 s, which matches the corresponding O rise of ~0.7 s in 500 mM KCl (Figure 2 and Figure S3, Table 1). This indicates that M and O intermediates appear sequentially in the K^+^-dependent photocycle, similar to the mode of Na^+^-dependent photocycle. 

**Figure 8:**
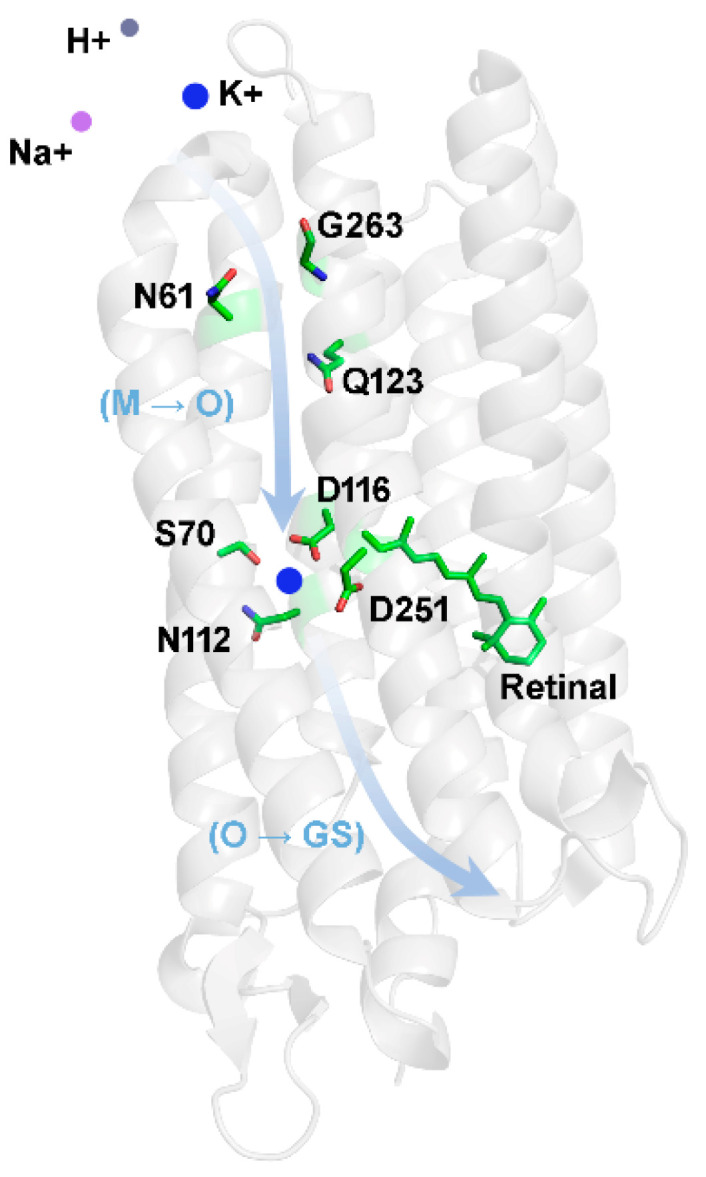
Tentative scheme for K^+^ transport by NdR2. The cytoplasmic ion uptake cavity is indicated by Gly263, Asn61, and Gln123. The Schiff base ion binding cavity is indicated by Asp116, Ser70, Asn112, Asp251, and retinal. Upon illumination, K^+^ (blue) competing with H^+^ (dark gray) and Na^+^ (purple) may be taken up through the cytoplasmic cavity, subsequently binding to the Schiff base cavity during M to O transition (indicated by M → O) and finally moving to the extracellular surface during the decay of O back to the initial NdR2 ground state (indicated by O → GS). An imaginary track of K^+^ is shown using the arrow line. Transmembrane helices are shown in grey. The model is based on the crystal structure of KR2 (PDB code: 6rew).

Based on our results and published data on Na^+^ transport, a tentative model for the transport of K^+^ in NdR2 is shown in Figure 8. In the ground state, the protonated Schiff base serves as a gate located between the cytoplasmic ion uptake cavity and the extracellular Schiff base cavity [14,15]. Upon illumination, all-*trans* retinal isomerizes to the 13-*cis* form [18,32], which results in the deprotonation of the Schiff base and the protonation of the counterion Asp116. This removes the energy barrier from the center of the ion transport pathway, probably during L/M intermediate [7,20,23,24]. As the cytoplasmic channel opens, K^+^ should be able to flow through the intracellular filter, which is surrounded by Gly263, Asn61, and Gln123. In this process, the transport of K^+^ may compete with that of H^+^ and Na^+^. Subsequently, K^+^ binds to the Schiff base cavity consisting of Asp116, Ser70, Asn112, and Asp251, which is also the binding site for Na^+^ [29,32]. The positive charge of K^+^ may force the protonated Asp116 to return the proton to the Schiff base, which in turn prevents the backflow of K^+^. In other words, the binding of K^+^ to the Schiff Base cavity during M decay is coupled to the deprotonation of Asp116 and the re-protonation of the Schiff Base. Therefore, as the concentration of K^+^ increases, the rate of K^+^ moving to this site also increases, leading to the acceleration of M decay. The accumulation of a red-shifted O intermediate (Figure 3 and Figure S3) confirms the binding of K^+^ at a later stage of the photocycle. Finally, K^+^ moves from the Schiff base cavity to the extracellular surface during the decay of the O intermediate.

### 3.2. Engineering of K^+^-Pumping Rhodopsin

Engineered light-controlled K^+^ pumps from NaR are promising in the expansion of scope of optogenetic applications. Modification at Gly263 and Asn61 in the cytoplasmic ion uptake cavity created pumps that can transport larger monovalent cations, like K^+^ and Cs^+^ [14,15,39]. Therefore, Konno et al. concluded that cations larger than Na^+^ could not permeate through the narrow cytoplasmic selectivity filter unless the introduction of large residues modified the local structure and increased the size of the filter. This, however, has been questioned by the fact that a dramatically reduced ion uptake cavity, rather than an enlarged one, as would be expected for K^+^ permeability, was discovered in the crystal structure of G263F [40]. These studies have suggested that the cytoplasmic filter might not follow the proposed size-exclusion mechanism to select K^+^. However, the cavity dimension of the G263F mutant may not be directly linked to rhodopsin’s ability to transport K. It is possible to have a large cavity capable of binding K, but the pump could be trapped in this state and unable to proceed further in transporting it across the membrane. It is well known that K^+^ needs to be dehydrated when entering K^+^ pumps or channels [50,51,52]. Most KR2-modified K^+^ pumps have been achieved by the substitution of Gly263 or Asn61 with Trp, Phe, or Leu at the entrance to the cytoplasmic ion uptake cavity. Thus, a possible interpretation is that these large hydrophobic residues might facilitate the transport of K^+^ by accelerating the dehydration uptake of K^+^, or by preventing the backflow of K^+^.

In this study, we compared the kinetics and functional activities of G263W/N61P to the WT. The residues of G263 and N61 appear conserved in the NaR family, and the amino acid sequence numbers (G263 and N61) for NdR2 are the same as KR2 (Figure S7). Based on the fact that KR2-G263W/N61P is capable of pumping K^+^, we hypothesized that NdR2-G263W/N61P could also pump K^+^ in high KCl, and we refer to it as a putative K^+^-pumping mutant. Previous studies have been limited to reporting the activity of G263W/N61P, lacking in-depth investigations into its underlying mechanisms [14,15,39]. We present, for the first time, the kinetic findings of K^+^ uptake of the mutant. Interestingly, we found that G263W/N61P mutant demonstrated a significant decrease in k_H_/k_K_ along with an increase in k_H_/k_Na_ (Figure 7). Not only would the uptake of K^+^ accelerate but also the uptake of Na^+^ would slow down when the selectivity for K^+^ was improved in G263W/N61P. This finding is in line with the corresponding activity assay in KR2 mutant studies, which shows increased pump activity of K^+^ and decreased pump activity of Na^+^ [14]. Therefore, we propose that the changes in both the relative rate of K^+^ uptake and that of Na^+^, need to be taken into account when screening efficient K^+^ pumps. In addition, our current experiments primarily assess changes in cytoplasmic ion selectivity (k_H_/k_K_) during ion uptake. There may be additional possible mechanisms for the observed increased K^+^ pumping ability of the G263W/N61P mutant, including changes in the affinity of K^+^ binding sites within the mutant’s interior or alterations in the kinetics of K^+^ release at the extracellular surface.

Notably, KR2 mutant S254A and eKR2, which have been modified in the vicinity of retinal and extracellular terminal, respectively, also have exhibited K^+^ pump activity [37,40]. In addition, KR2 mutant R109Q, whose extracellular half-channel has been modified, showed passive preferential leakage for K^+^ [53]. These imply that a second ion selectivity filter, at least, may exist inside the extracellular half-channel or other sites in addition to the cytoplasmic filter. Thus, future modifications focusing on extracellular ion pathways might further improve the ion selectivity for K^+^ by accelerating the release of K^+^.

To evaluate the ion selectivity and screen for K^+^ pumps, traditional cell-based pump activity assays have been widely applied [14,39,40]. However, high batch-to-batch variation in protein expression level, cell viability, and intrinsic low activity of K^+^ transport have made it difficult to screen for promising candidates for K^+^ pumps. Distinct from the conventional activity assay, we introduced kinetic analysis of competitive ion uptake and the analysis of photocurrent into the study of K^+^ transport, specifically the determination of K_H_/K_K_. This provides methodological assistance for applications such as screening efficient K^+^ pumps and the engineering of microbial rhodopsins. It should also be noted that this fundamental biophysical research has the potential for wide-ranging impacts in various fields, including medicine [54], chemistry [55], biology [56], microfluidics [57,58,59], and other related areas.

The method of directly titrating the sample with KCl to estimate the value of k_H_/k_K_ has its limitations. When we conducted the direct titration of the WT in 1 M NMG-Cl with KCl, we obtained only a fraction of the typical sigmoidal-shaped titration curve dataset, from which k_H_/k_K_ could not be estimated. As demonstrated by Kato et al. [33] and our own NaCl titration curve (Figure S4), NaCl concentrations need to be at least 10 mM to obtain a typical sigmoidal-shaped titration curve dataset, from which k_H_/k_Na_ could be directly estimated. Given that the value of k_H_/k_Na_ is ~2.6 × 10^4^, the value of k_Na_[Na^+^] is approximately 38 times higher than that of k_H_[H^+^] under conditions of 10 mM of [Na^+^] and pH 8.0. This indicates that Na^+^ uptake in 10 mM NaCl has the potential to completely inhibit H^+^ uptake, leading to a full sigmoidal-shaped titration curve. Likewise, K^+^ uptake competes with H^+^ uptake in the direct titration experiment using KCl. To achieve a typical sigmoidal-shaped titration with KCl, where we assume that k_k_[K^+^] should also be approximately 38 times higher than k_H_[H^+^], the concentration of K^+^ would need to be around 122 M, given the value of k_H_/k_K_ ~ 3.2 × 10^8^. At the concentration of a saturated solution of KCl (~4.0 M), the value of k_k_[K^+^] is only slightly higher than that of k_H_[H^+^]. This indicates that even at the maximum solubility concentration, K^+^ uptake is unable to completely inhibit H^+^ uptake in the WT. As a result, it becomes challenging to obtain a sigmoidal-shaped titration curve through direct titration using KCl. Therefore, rather than conducting direct titration with KCl, we performed the indirect titration on the WT sample in 1 M KCl using NaCl (Figure 6a). In this setup, the uptake of Na^+^, K^+^, and H^+^ compete with each other, allowing us to estimate k_H_/k_K_ based on Equation (2). Furthermore, it is worth noting that the ratio of the rate constant of K^+^ uptake to that of other ions does not directly represent the relative permeability of K^+^ across the membrane but rather across the half-channel on the cytoplasmic side. A more direct and sensitive method, like potassium isotopes, would be desirable.

## 4. Materials and Methods

### 4.1. Gene Cloning, Protein Expression, and Purification

The cloning, protein expression, and purification of the *N. dokdonensis* DSW-6 gene (GI: 442800350) [8] are described elsewhere with some modifications [44]. Briefly, NdR2 expression was induced with 1 mM IPTG (Amresco, Allentown, PA, USA) and 5 μM all-*trans* retinal (Sigma, St. Louis, MO, USA) at 37 °C for 3 h. After being disrupted via sonification, the cell debris was removed via centrifugation at 4800× *g* for 15 min at 4 °C. The collected cell lysate was centrifuged at 20,000× *g* for 70 min at 4 °C to isolate the membrane fraction, which was subsequently solubilized in a n-dodecyl-D-maltoside (DDM, Anatrace, Maumee, OH, USA) solution with 10 mM Tris-HCl, 400 mM KCl, 20 mM imidazole, and 1% DDM at pH 8.0. After the undissolved membrane was removed by centrifugation at 20,000× *g* for 50 min at 4 °C, we loaded the micelle solution on a Ni-NTA agarose column (Qiagen, Germantown, MD, USA), followed by elution buffer with 10 mM Tris-HCl, 400 mM KCl, 500 mM imidazole, and 0.1% DDM at pH 8.0. Finally, we dialyzed the purified protein in the desired solution for later experiments. For the photocurrent measurements, we reconstituted purified NdR2 into liposomes at a mole ratio of ~1:50, as previously reported [44].

### 4.2. Flash Photolysis

We collected UV–vis absorption spectra with a UV-2550 spectrometer (Shimadzu, Kyoto, Japan). We measured the kinetics of the NdR2 photocycle via time-resolved difference spectroscopy at specific wavelengths. Absorbance changes at 525, 410, and 610 nm reflected the kinetics of ground state, M intermediate, and O intermediate, respectively. The probe light was focused on the sample and then collected by a photomultiplier tube (R10699, Hamamatsu Photonics, Hamamatsu, Shizuoka Prefecture, Japan) [44]. Kinetic traces were recorded with an oscilloscope (MSO 44, Tektronix, Beaverton, OR, USA). Samples were excited by a 10 W xenon flash lamp (~10 μs duration) covered with a long pass filter (>490 nm). The recorded kinetics was the same as the published photocycle initiated using a 532 nm nanosecond laser [41]. No two-photon excitation was detected. The kinetic data were fitted with the multi-exponential function using OriginPro 2018. We performed all measurements at about 24.5 °C. We conducted all experiments at pH 8.0 to avoid the partial protonation of Asp116, which occurs at acidic pH and produces a mixture of two conformations [7]. It should be noted that we derived the lifetime and error values by fitting the time-resolved data from a single flash photolysis experiment. This is because it is common in time-resolved spectroscopy to report lifetimes from one experiment rather than an average from multiple experiments.

Na^+^, H^+^, and K^+^, competing with each other, are taken up from the cytoplasmic surface during the M decay. As the O forms, they translocate across the retinal Schiff base and bind to the putative site near Asp116, thereby forming Na^+^-bound O state (O_Na_), H^+^-bound O state (O_H_), and K^+^-bound O state (O_K_), respectively. According to the parallel/competing reaction proposed by Kato et al. [33], the simplified scheme of M intermediate transiting irreversibly to O intermediate is shown below:

$$M\overset{k_{Na}}{\to }O_{Na};\;M\overset{k_{H}}{\to }O_{H};\;M\overset{k_{K}}{\to }O_{K}$$

k_H_, k_Na_, and k_K_ refer to the rate constant of H^+^ uptake, Na^+^ uptake, and K^+^ uptake, respectively. Accordingly, the instantaneous M concentration, [M], derived from the M to O transition rate equation, is demonstrated as Equation (1). [M]_0_ is the initial concentration of M before its decay. The corresponding lifetimes of M decay were plotted against [Na^+^] to quantitatively investigate the ion selectivity (k_H_/k_Na_ and k_H_/k_K_) using Equation (2).

(1) $$\left[M\right]={\left[M\right]}_{0}\exp(-\left(k_{H}\left[H^{+}\right]+k_{Na}\left[{Na}^{+}\right]+k_{K}[K^{+}]\right)t)$$

(2) $$\tau_{1/2}=\frac{ln2}{k_{H}\left[H^{+}\right]+k_{Na}\left[{Na}^{+}\right]+k_{K}[K^{+}]}$$

### 4.3. Photocurrent Measurements of the Light-Driven Ion Transport Activity

The photoelectric response of lipid-reconstituted NdR2 followed the published protocol but with some modifications [45,60]. Indium tin oxide (ITO) glass was washed successively with acetone, ethanol, and water via ultrasonic bath. Lipid-reconstituted NdR2 was air-dried and immobilized on the surface of ITO glass. We measured the photocurrent by connecting the protein-coated working electrode to the positive input and the reference electrode to the negative input of the current amplifier (SR570, Stanford Research Systems, Sunnyvale, CA, USA). The output signal was recorded using a digital oscilloscope (DPO2002B, Tektronix, Beaverton, OR, USA). The sample was illuminated with a halogen lamp (150 W) equipped with a 525 ± 39 nm band pass filter and a hot mirror. We introduced modulation of the continuous-wave light using a beam shutter (SH1, Thorlabs, Newton, NJ, USA) with a rise time of about 10 ms. 

For the measurement of the apparent pump activity in *E. coli* cells, we assembled the sample chamber in the following order: ITO glass, cell suspension, dialysis membrane, electrolyte solution, and another ITO-coated glass. Before being injected into the sample chamber, *E. coli* cells expressing NdR2 were washed three times and equilibrated in the desired salt solution. We resuspended cells in the solution containing 50 μM carbonylcyanide-m-chlorophenylhydrazone (CCCP) as needed [7,44]. The stationary photocurrent was collected as a direct current (DC) signal for the pump activity assay. Over-expression of a foreign membrane protein NdR2 seemed to make the host so vulnerable that *E. coli* cells could hardly survive the treatment with 1 M salt solution. We could detect neither H^+^ transport nor Na^+^ transport activity. Thus, as a trade-off, we decreased the salt concentration to 500 mM.

## 5. Conclusions

In this study, we report an unexpected K^+^-dependent photocycle kinetics and photocurrent, which suggest the uptake of K^+^ by WT NdR2. K^+^ may be taken up through the cytoplasmic cavity during the M to O transition, competing with Na^+^ or H^+^ in the photocycle. Our findings provide novel kinetic insights into the K^+^ selectivity and transport mechanism. Furthermore, Na^+^-pumping microbial rhodopsins are the next-generation tools of optogenetics. Our studies on NdR2 may have practical implications in screening efficient light-driven K^+^ pumps and guiding the design of other optogenetic tools for neuroscience.

## Figures and Tables

**Table 1:** Lifetimes of M decay for the WT NdR2 under various concentrations of KCl.

[KCl], mM	τ_1_, s (K^+^-Dependent)	τ_2_, s (K^+^-Independent)
0 *	-	2.08 ± 0.04
100	0.92 ± 0.14	2.39 ± 0.44
500	0.60 ± 0.08	2.02 ± 0.17
1000	0.35 ± 0.05	1.99 ± 0.09

* When KCl was absent, 1 M NMG-Cl was provided as background salt to maintain sufficient ionic strength.

## Data Availability

Data is contained within the article or supplementary material.

## Supplementary Materials

The following supporting information can be downloaded at: https://www.mdpi.com/article/10.3390/ijms241914414/s1.

## Abbreviations

NdR2	*Nonlabens dokdonensis* rhodopsin 2
BR	bacteriorhodopsin
KR2	*Krokinobacter eikastus* rhodopsin 2
HR	halorhodopsin
NaR	Na^+^-pumping rhodopsin
IPTG	Isopropyl-β-D-thiogalactopyranoside
DDM	n-dodecyl-β-D-maltoside
NMG	N-methyl-D-glucamine
CCCP	carbonylcyanide-m-chlorophenylhydrazone
ITO	indium tin oxide
DC	direct current
WT	wild type
