# Supplementary material for: K+-Dependent Photocycle and Photocurrent Reveal the Uptake of K+ in Light-Driven Sodium Pump
Source: Int J Mol Sci. 2023 Sep 22;24(19):14414. doi: 10.3390/ijms241914414 (PMC10572131; doi:10.3390/ijms241914414)
Supplement: Supplementary file 1 [file ijms-24-14414-s001.zip › ijms-2593469-supplementary.pdf]

Supplementary materials

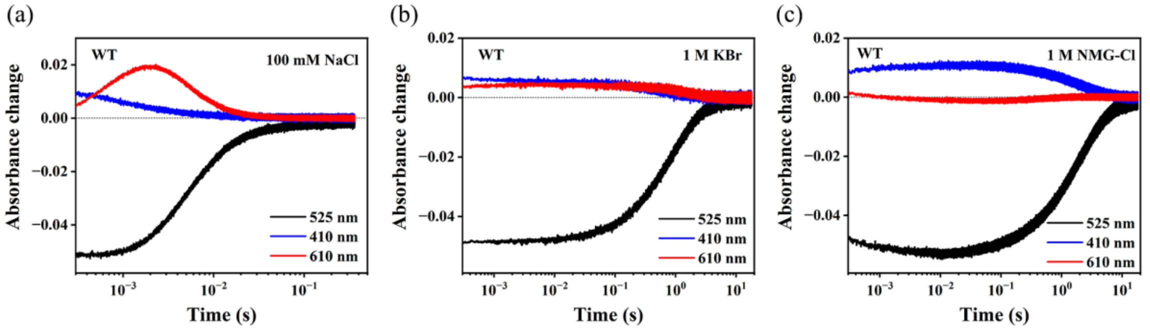

Figure S1. Photocycle kinetics of WT NdR2 at pH 8.0. Absorbance changes of the WT in 0.05% DDM solution containing (a)100 mM NaCl, (b) 1 M KBr, and (c) 1 M NMG-Cl are shown at characteristic wavelengths of 525 nm, 410 nm, and 610 nm.

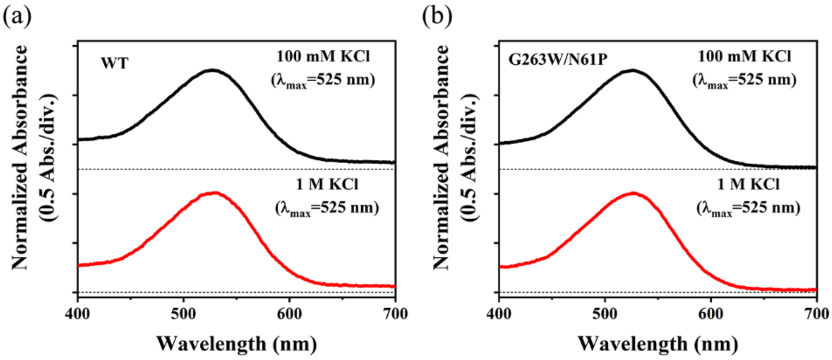

Figure S2. Characteristic absorption spectra of NdR2 at pH 8.0. (a) the WT and (b) G263W/N61P are in 100 mM and 1 M KCl solution, respectively.

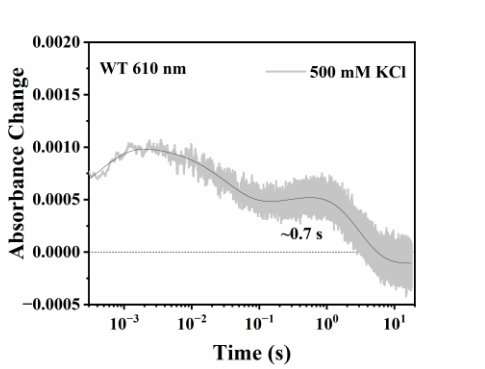

Figure S3. O intermediate kinetics of NdR2 at pH 8.0. Absorbance changes of the WT in 500 mM KCl solution containing 0.05% DDM, were measured at 610 nm. The fitting line is superimposed on the raw data. The time constant of the rise of the O intermediate is indicated.

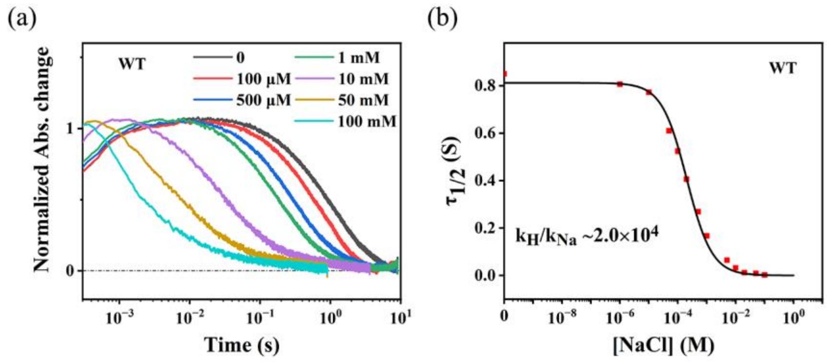

Figure S4. Kinetic analysis of competitive uptake of Na^+ and H^+ during M decay of WT NdR2 at pH 8.0. The WT in 1M NMG-Cl solution was titrated with 4 M NaCl. (a) Normalized absorbance changes at various concentrations of NaCl detected at 410 nm. (b) The $\tau_{1/2}$ of M intermediate against NaCl concentrations (0-100 mM). The solid curve represents the fitting result corresponding to Equation 2. The calculated value of k_H/k_{Na} is shown accordingly.

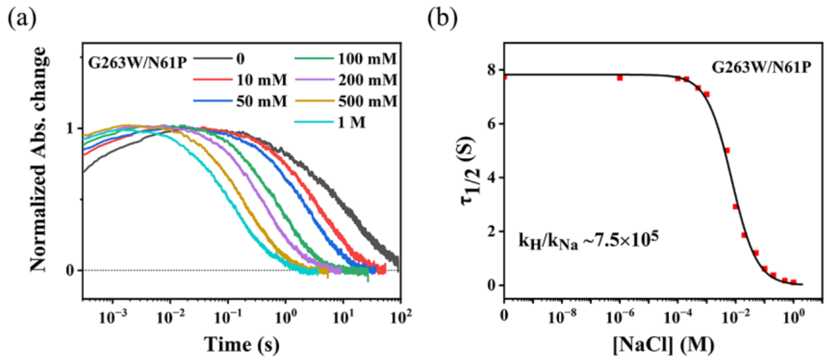

Figure S5. Kinetic analysis of competitive uptake of Na^+ and H^+ during M decay of G263W/N61P at pH 8.0. The mutant in the background salt solution of 50 mM NMG-Cl was titrated with 4 M NaCl. (a) Normalized absorbance changes at various concentrations of NaCl were obtained. (b) Calculated $\tau_{1/2}$ of M intermediate was plotted against NaCl concentrations. The solid curve represents the fitting result corresponding to Equation 2. The calculated value of k_H/k_{Na} is shown accordingly.

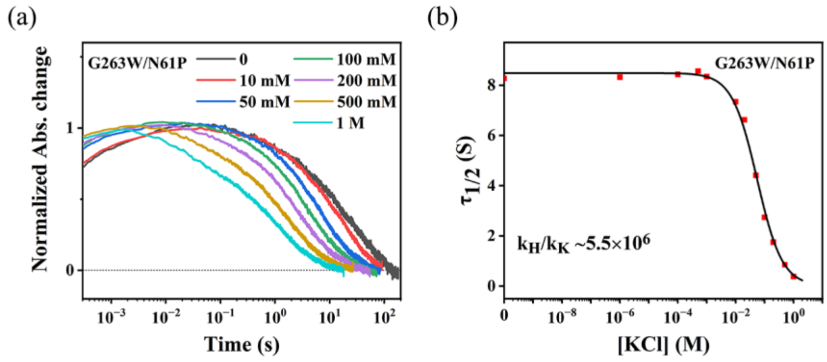

Figure S6. Kinetic analysis of competitive uptake of K^+ and H^+ during M decay of G263W/N61P at pH 8.0. The mutant in the background salt solution of 50 mM NMG-Cl was titrated with 3 M KCl. (a) Normalized absorbance changes at various concentrations of KCl were obtained. (b) Calculated $\tau_{1/2}$ of M intermediate was plotted against KCl concentrations. The solid curve represents the fitting result corresponding to Equation 2. The calculated value of k_H/k_K is shown accordingly.

```
Halobacterium salinarum NRC-1 (BR)    -MLELLPTAVEGVS-----QAQITGRPEWIWLALGTALMGLGTLYFLVKG   44
Dokdonia eikasta (KR2)                MTQELGNANFENFIGATEGFSEIAYQFTSHILTLGYAVMLAGLLYFILT-   49
Nonlabens dokdonensis DSW-6 (NdR2)    MIQDLGNSNFENYVGATDGFSEMAYQMTSHVLTLGYAVMFAGLLYFILT-   49
Indibacter alkaliphilus LW1 (IaNaR)   -MENLGNATFENYIGLQDGFNEMAYQMVAHVLTLGYAVMLAGLFYFVLT-   48
Dokdonia sp. PRO95                    MTQELGNANFENFIGATEGFSEIAYQFTSHILTLGYAVMLAGLLYFILT-   49
Gillisia limnaea (GLR)                MTQELGNANFENFIGATEGFSEIAYQFTSHILTLGYAVMLAGLLYFILT-   49
Nonlabens marinus S1-08 (NM-R2)       -MQQLGNSNFENYIGASEGFSEMAYQMTSHVLTLGYAVMLAGLLYFILT-   48
Jannaschia seosinensis (JsNaR)        ------MPNYENFF----AFEYWQYEVVRHMFAFTAAVFAAAFVYFALT-   39

Halobacterium salinarum NRC-1 (BR)    MGVSDPDAKKFYAITTLVPAIAFTMYLSMLLGYGLTMVP-------FGGE   87
Dokdonia eikasta (KR2)                IKNVDKKFQMSNILSAVVMVSAFLLLYAQAQNWTSSFTFNEEVGRYFLDP   99
Nonlabens dokdonensis DSW-6 (NdR2)    IKNVDKKYRMSNILSAVVMVSAALLLYAQAGNWTESFAFDAERGKYFLVE   99
Indibacter alkaliphilus LW1 (IaNaR)   IKTVAPRFRTSSVLSVVVMVSAFLLLYVQASNWTESFVFDTERGKYFLGE   98
Dokdonia sp. PRO95                    IKNVDKKFQMSNILSAVVMVSAFLLLYAQAQNWTSSFTYNEEVGRYFLDP   99
Gillisia limnaea (GLR)                IKKVDKKYQMSNILSAVVMVSAFLLLYAQAENWTTSFTFDISRGKYFLEP   99
Nonlabens marinus S1-08 (NM-R2)       IKKVDKRFQMSNILSAVVMVSAFLLLYAQAGNWTSSFTFDIELGRYFLDP   98
Jannaschia seosinensis (JsNa)         MRQVAERYRLASIISCVVMISATLELFLLWLMWNRAFEFDITTMTYARAE   89

Halobacterium salinarum NRC-1 (BR)    QNPIYWA--RYADWLFTTPLLLLDLALLVDADQGTILALVG----ADGIM   131
Dokdonia eikasta (KR2)                SGDLFNNGYRYLNWLIDVPMLLFQILFVVSLTTSKFSSVRNQFWFSGAMM   149
Nonlabens dokdonensis DSW-6 (NdR2)    GGDLFNNGYRYLNWLIDVPMLLFQILFVVQLTKSKLSSVRNQFWFSGAMM   149
Indibacter alkaliphilus LW1 (IaNaR)   GNDLFNNGYRYLNWLIDVPMLLFQILFVVTLTKSNFSSIRNQFWISGTGM   148
Dokdonia sp. PRO95                    SGDLFNNGYRYLNWLIDVPMLLFQILFVVSLTSSKFSSVRNQFWFSGAMM   149
Gillisia limnaea (GLR)                NGDLFNNGYRYLNWLIDVPMLLFQILFVVSLTKSKFSSIRNQFWFSGAMM   149
Nonlabens marinus S1-08 (NM-R2)       DGDLFNNGYRYLNWLIDVPMLLFQILFVVTLTKSKLSSIRNQFWFSGTMM   148
Jannaschia seosinensis (JsNaR)        G-FIFANGYRYANWMIDVPMLMTQLLVVLGFAGRDMLSRWWKLMALGVFM   138

Halobacterium salinarum NRC-1 (BR)    IGTGLVGALTKVYSYR---------FVWWAISTAAMLYILYVLFFGFTSK   172
Dokdonia eikasta (KR2)                IITGYIGQFYEVSNL-------TAFLVWGAISSAFFFHILWVMKKVINEG   192
Nonlabens dokdonensis DSW-6 (NdR2)    IITGYIGQYYEVTDL-------SAFFIWGAISTVFFFHILWLMNKVIKEG   192
Indibacter alkaliphilus LW1 (IaNaR)   IVTGYIGQFYEVTDL-------TMFAIWGAISTVFFFHILWLMKKVIDEG   191
Dokdonia sp. PRO95                    IITGYIGQFHEVSNL-------TAFLVWGAISSAFFFHILWVMKKVIKEG   192
Gillisia limnaea (GLR)                IITGYIGQFYEVSNL-------TAFFVWGAISSVFFFHILWVMKKVINEG   192
Nonlabens marinus S1-08 (NM-R2)       IITGYIGQFYEVSDI-------TWFLIWGAISTVFFFHILYLMKKVINEG   191
Jannaschia seosinensis (JsNaR)        ILTAYIGQYYEPQAAGIIDESAFGFWFWGIVSWIAFLVLLWLLYKNVEEG   188

Halobacterium salinarum NRC-1 (BR)    AESMRPEVASTFKVLRNVTVVLWSAYPVVWLIGSEG------AGIVPLNI   216
Dokdonia eikasta (KR2)                KEGISPAGQKILSNIWILFLISWTLYPGAYLMPYLTGVDGFLYSEDGVMA   242
Nonlabens dokdonensis DSW-6 (NdR2)    KVGIPKKGQKILSNIWILFLVSWFLYPGAYLMPHLGGIEGFLFNESGVVG   242
Indibacter alkaliphilus LW1 (IaNaR)   KDGIPAKAQETLQSIWVLFLVSWMLYPGAYLMPHLAGIEGLFFSEIGVVA   241
Dokdonia sp. PRO95                    KEGISPAGQKILSNIWILFLISWTLYPGAYLMPYLTGVDGFLYSEDGVMA   242
Gillisia limnaea (GLR)                KEGLSADAQKILSNIWVLFLVSWFLYPGAYLMPYLTGLDGFFFSEDGVMA   242
Nonlabens marinus S1-08 (NM-R2)       KEGISTKGQKILSNIWILFLISWFLYPGAYLMPYLGGIDGFLYNESGVVG   241
Jannaschia seosinensis (JsNa)         RARMGGEASKLMGAAWTLMWITWTIYGLVYLVPGIPGIN---ESSTWIVI   235

Halobacterium salinarum NRC-1 (BR)    ETLLFMVLDVSAKVGFGLILLRSRAIFGEAEAPEPSAGDGAAATSD   262
Dokdonia eikasta (KR2)                RQLVYTIADVSSKVIYGVLLGNLAITLSKNKELVEANS--------   280
Nonlabens dokdonensis DSW-6 (NdR2)    RQITYTIADVCSKVIYGVLLGNLALVLSKNKEMIETA---------   279
Indibacter alkaliphilus LW1 (IaNaR)   RQITYTIADVSSKVIYGILLTNVAQVMSKEEGYLEHTT--------   279
Dokdonia sp. PRO95                    RQLVYTIADVCSKVIYGVLLGNLAITLSKNKELVEANS--------   280
Gillisia limnaea (GLR)                RQLTYTIADVCSKVIYGVLLGNLALKLSNNKEMVELSN--------   280
Nonlabens marinus S1-08 (NM-R2)       RQITYTIADVCSKVIYGVLLGNLAMTLSTKNKNEHKPA--------   279
Jannaschia seosinensis (JsNaR)        RQGGYTFADVTSKAVFGVILSYTAMTLSKEPERNTTRKAA------   275
```

Figure S7. Sequence alignment of amino acids of BR, KR2, NdR2, IaNaR, Dokdonia sp. PRO95, GLR, NMR2, and JaNaR. The sequences were aligned using the ClustalW algorithm implemented in the Mega X software.
